# Metatranscriptomic Analyses Reveal the Functional Role of *Botrytis cinerea* in Biochemical and Textural Changes during Noble Rot of Grapevines

**DOI:** 10.3390/jof8040378

**Published:** 2022-04-08

**Authors:** Ádám István Hegyi, Margot Otto, József Geml, Júlia Hegyi-Kaló, József Kun, Attila Gyenesei, Rian Pierneef, Kálmán Zoltán Váczy

**Affiliations:** 1 Food and Wine Research Institute, Eszterházy Károly Catholic University, H-3300 Eger, Hungary; hegyi.adam@uni-eszterhazy.hu (Á.I.H.); geml.jozsef@uni-eszterhazy.hu (J.G.); hegyi-kalo.julia@uni-eszterhazy.hu (J.H.-K.); 2 ELKH-EKKE Lendület Environmental Microbiome Research Group, Eszterházy Károly Catholic University, H-3300 Eger, Hungary; margot.27.muller@gmail.com; 3 Genomics and Bioinformatics Core Facility, University of Pécs, H-7601 Pécs, Hungary; kun.jozsef@pte.hu (J.K.); gyenesei.attila@pte.hu (A.G.); 4 Department of Pharmacology and Parmacotherapy, University of Pécs Medical School, H-7624 Pécs, Hungary; 5 Biotechnology Platform, Agricultural Research Council-Onderstepoort Veterinary Research, Pretoria 0110, South Africa; pierneefr@arc.agric.za

**Keywords:** *Botrytis cinerea*, WGCNA clustering, differential expression, enrichment, noble rot, secondary metabolites

## Abstract

*Botrytis cinerea*, can lead to the formation of noble rot (NR) of grape berries under certain environmental conditions, resulting in favored metabolic and physical changes necessary for producing highly regarded botrytized wines. The functional genes involved in the textural and biochemical processes are still poorly characterized. We generated and analyzed metatranscriptomic data from healthy (H) berries and from berries representing the four stages of NR from the Tokaj wine region in Hungary over three months. A weighted gene co-expression network analysis (WGCNA) was conducted to link *B. cinerea* functional genes to grape berry physical parameters berry hardness (BH), berry skin break force (F_sk), berry skin elasticity (E_sk), and the skin break energy (W_sk). Clustered modules showed that genes involved in carbohydrate and protein metabolism were significantly enriched in NR, highlighting their importance in the grape berry structural integrity. Carbohydrate active enzymes were particularly up-regulated at the onset of NR (during the transition from phase I to II) suggesting that the major structural changes occur early in the NR process. In addition, we identified genes expressed throughout the NR process belonging to enriched pathways that allow *B. cinerea* to dominate and proliferate during this state, including sulphate metabolizing genes and genes involved in the synthesis of antimicrobials.

## 1. Introduction

Noble rot (NR) of grape berries is a unique oenological process characterized by *Botrytis cinerea* infecting of fully ripened berries resulting in naturally fermented sweet wines [1]. Amongst several textural, color, and biochemical changes, these berries have an increased sugar concentration as well as a distinct aromatic profile which is the reason botrytised wines are called “the wine of kings, the king of wines” [2,3]. In addition to causing NR, *B. cinerea* can, however, also cause grey rot (GR), a condition rendering grape berries unusable for wine production, resulting in significant economic losses [4].

The beneficial state of *B. cinerea*, which is also called “Dr. Jekyll and Mr. Hyde fungus” [5], appears rarely and is found in low abundance in several wine regions that have a suitable meso-climate such as regions in France (Sauternes AOC in Bordeaux and Selection de Grains Nobles), areas in the Rheingau region of Germany (Trockenbeerenauslese wines), Austria (Burgenland wines) and *Tokaj aszú* wines in Hungary [1,6,7,8]. *B. cinerea* can initiate significant changes in the aromatic precursor composition of grape juice with its secondary metabolites, giving it a unique sensory profile differentiated by the level of botrytiziation [9], the ratio of NR/GR berries [10,11], and by the production region (terroir) [5].

The production process of botrytized wines begins with manual harvest of NR grape berries, with healthy, NR and GR grape berries that are present in the same field. Noble rotten berries are soft and succulent, NR berries have a soft, but stable structure, which allows them to hang on the stems for an extended period during which dehydration occurs, giving the berry its characteristic NR appearance [1,9]. Conversely, GR berries have a decomposed appearance and disintegrate easily [9,12,13,14]. The NR process occurs in four phases, resulting in berries with appropriate texture and color for further processing [9,15] During infection, several changes are observed in grape berries, including water evaporation which leads to increased sugar and citric acid concentration. In addition, the cell wall is degraded, causing tissue senescence and resulting in further moisture loss and textural changes [16,17,18].

Over the past few years, texture measurements such as puncture and compression tests including measurements of grape berry hardness (B_H), berry skin break energy (W_sk), the berry skin break force (F_sk), and the berry skin elastic modulus (E_sk) have been used to assess the quality evolution of grapes during ripening. Texture measurements have proven to be a useful tool to discriminate berries from different red varieties, parcels, and quality [18,19,20]. Maury et al. (2009) defined nine different ripening stages for Cabernet franc berries in 2005 and 2006 and thus could be a useful tool to characterize berries infected with noble rot [19]. Therefore, texture measurements could be an important tool for characterizing berries undergoing NR. Differentiation of NR berries is currently still subjective. For example, Carey et al. (2004) [20] defined 2 NR stages, using color, presence of fungal growth, and level of dehydration (thus texture) as distinguishing characteristics.

Studies concerning NR have mostly investigated the role of climate change [21,22] (e.g., temperature, humidity, and precipitation) favored harvest times [9] and viticulture elements [5,23] on the NR process. It was found that morning fog and dew formation and warm sunny days [1,2] favor the onset of NR in berries with desirable sensory parameters (e.g., sugar level, acid level, sugar-acid balance) and that the optimal season for botrytization is late autumn [9]. There have been very few gene expression studies focusing on the role of *B. cinerea* in NR development, and these mostly included postharvest semi-artificial NR. For example, a recent study analyzed differentially expressed genes (DEGs) of postharvest *Garganega* berries and an open access database of in vitro *B. cinerea* cultures isolated from *Marsalan* cultivar [24]. Consequently, we lack the conceptual understanding of what functional genes of *B. cinerea* are expressed in different phases of naturally occurring NR in vineyard conditions and how these contribute to the physico-chemical changes of grape berries and to the properties of botrytized wines.

In the present study, we generated metatranscriptomic data from healthy (H) berries and from berries representing the four stages of NR collected throughout the three-month harvest season in the Tokaj wine region of Hungary. We performed differential expression analyses (DE) and pathway enrichment (PE) combined with weighted gene co-expression network analyses (WGCNA) to investigate the role of *B. cinerea* in the four stages of NR in terms of textural changes and to a lesser extent biochemical changes that take place during NR. We hypothesized that (i) the expression of *B. cinerea* functional genes would be higher in NR than in healthy berries. We also hypothesized that (ii) there would be significant changes in the composition of functional genes among later phases of NR, as berry moisture content decreases. Because most of the pronounced physico-chemical changes occur at the onset of NR, i.e., during the transition from phase I to phase II [9], we hypothesized that (iii) genes involved biochemical pathways responsible for plant cell wall degradation to be up-regulated in phase II when NR development starts. Finally, because of the gradual decrease in temperature and increase in available moisture during the harvest period, we hypothesized that (iv) sampling time also influences the composition expressed functional genes, even within NR phases.

## 2. Materials and Methods

### 2.1. Sampling and RNA Extraction

The berry samples were collected in 2016, in the Betsek vineyard (48°11′18.6″ N 21°19′01.8″ E) located in Mád (Tokaj wine region, Hungary), where the prevalence of NR berries is higher than in other surrounding vineyards. The vineyard was planted in 1984 and has a south to northwest position with an inclination of 5–20%. The main soil type in this region is yellow clay which consists of yellow and white tuff. Grape berries were collected from Furmint cultivar in September, October, and November respectively. Sampling was performed based on four visually distinguishing phases in terms of color, presence of fungal growth, and textural changes. The phases were defined as follows: (I) Fully mature, healthy, green grape berries with no sign of fungal growth or textural change; (II) mature grape berries that start undergoing a color change, showing yellow of brown spots and have first signs of fungal growth but do not show any change in texture; (III) grape berries have a dark purple color, more prevalent fungal growth than the previous stage, but do not show signs of dehydration; (IV) grape berries are dark purple in color, are riddled with fungal growth, and have a soft, shriveled, and dehydrated texture [9]. Five replicate samples were collected from healthy grape berries and grape berries showing the four distinctive NR phase (I, II, III, and IV) characteristics as previously described from each respective month resulting in a total of sixty samples. It is important to note that in terms of berry texture, the process of noble rot on grape vines has many similarities to that of dried grapes, where *B. cinerea* infection may also be present [25]. The collected grape berries were placed in 50 mL tubes which were subsequently placed in liquid nitrogen. The collected samples were immediately transported to the analytical lab at the Food and Wine Research Institute (Eszterházy Károly Catholic University) and were stored at −80 °C until RNA extraction. A protocol optimized for extraction of RNA from grapevine tissue by Reid et al. (2006) [26] was used for RNA extraction. Powdered sample (5 g) was weighed and placed into a 50 mL centrifuge tube to which 20 mL of extraction buffer [300 mM Tris HCL (pH 8.0), 25 mM EDTA, 2 M NaCl, 2% CTAB, 2% PVPP, 0.05% spermidine trihydrochloride] was added. The tubes were then incubated at 65 °C for ten minutes during which they were briefly vortexed every second minute. Thereafter, an equal volume of isoamyl alcohol (24:1) was added and the tubes were vortexed briefly and centrifuged at 4800 g for 10 min at 4 °C. The supernatant was then transferred to a new 50 µL Eppendorf tube and the centrifugation step was repeated. This was followed by transferring 10 µL of the supernatant into a new 50 mL Eppendorf tube and the addition of a tenth of the volume of 3 M sodium acetate buffer (pH = 5.2) and 0.6 volumes of ice-cold isopropanol. The samples were then incubated for 30 min at −70 °C and then centrifuged at 4800 g for 30 min at 4 °C. The supernatant was then discarded, and the remaining solution was washed with 5 mL of 70% ethanol and centrifuged at 4800 g for 15 min. The resulting supernatant was discarded, and the precipitate was lyophilized. The dried samples were subsequently dissolved in 1 mL of TE (pH = 7) buffer and transferred to a new 2 mL Eppendorf tube. Thereafter, 300 µL of 8 M LiCL was added and the samples were incubated overnight at 4 °C. The following day, the samples were centrifuged at 21,000 g for 30 min at 4 °C followed by a washing step with 70% of ice-cold ethanol. The resulting supernatant was discarded, and the precipitate was dried with a lyophilizer. The resulting pellets were dissolved in 42 µL of nuclease-free water. Finally, 2% of mercaptoethanol was added to the isolated RNA before its quality was measured on a nanodrop 1000 instrument (Thermo Fischer Scientific, Waltham, MA, USA). The extracted RNA was stored at −20 °C until sequencing. Single-end RNA sequencing was conducted on an Illumina HiScan sequencer at the UD GenoMed Medical Genomic institute at the University of Debrecen. The sequencing yielded 7.8–40.6 million reads per sample with a mean length of 76 bp.

### 2.2. Berry Skin Physical Parameter Measurement

For the berry texture profiling, commonly used testing methods were followed [27]. Operative conditions were chosen based on research results presented in the literature [28]. Berry skin break energy (W_sk), berry skin break force (F_sk), and berry skin elastic modulus (E_sk) were measured by puncture test, while berry hardness (BH) was measured by compression test, using instruments and probes described in Hegyi-Kaló et al. (2020) [9]. The distribution of the measured textural parameters can be found in Table 1.

### 2.3. RNAseq Data Analyses

The quality control of the RNAseq reads was assessed with FastQC v0.11.5 (Babraham Bioinformatics, Cambridge, UK). For the trimming of the reads Trimmomatic v0.36 was used to remove TruSeq2-PE adaptors with a simple clip threshold, an indrome threshold, and a seed mismatch threshold set at 10, 30, and 2 respectively [29]. In addition, the sequencing reads were filtered according to base call quality using the FASTX-TOOLKIT, specifically the fastq_quality_filter with parameters set at the following threshold: -Q33-q30 -p50. Then the filtered sequences were normalized according to these literatures [30,31] and Khmer v2.1.1 using the following command normalize-by-median.py parameters: -k20 -C20 -N4 -X 50e9. After, low abundance fragments of high-coverage reads were removed with Khamer and command filter-abund py -V. The resulting high-quality reads were aligned to the *B. cinerea* B05.10 reference genome [32] and split into their respective genes using an in-house Python script using Salmon version 1.3.0 [33]. After alignment, the transcript genes were linked to gene names using the GIF annotation for each genome using the GenomicFeatures package in R [34]. The DNA sequences have been deposited in NCBI under BioProject PRJNA736205 and BioSAMPLE SAMN19612984.

#### 2.3.1. Statistical Analyses

R (R Core Team, 2013) statistical software was used for all analyses. ANOVA and Tukey’s HSD test was performed to analyze the changes in the richness and abundance of all *B. cinerea* functional genes according to the four botrytization phases and three sampling dates. For the visualization of trends in the functional genes among phases and dates, non-metric multidimensional scaling (NMDS) analyses were used on all *B. cinerea* genes on median of ratios normalized abundance values, using the vegan package [35]. The data were subjected to 9999 iterations per run utilizing the Bray–Curtis dissimilarity and a random starting number. 

#### 2.3.2. Weighted Gene Co-Expression Analyses

For the observation of all *B. cinerea* functional genes, co-expression patterns with textural variables namely berry skin break force (F_sk), berry skin break energy (W_sk), berry skin elastic modulus (E_sk), and berry hardness (BH), weighted gene co-expression network analysis (WGCNA), using the WGCNA R package was performed [36]. Our analyses were run with 100 genes as minimum cluster size using Pearson correlation. The threshold power and merging threshold was set at 12 and 0.25 respectively. The clustering was visualized as a network cluster dendrogram. For the measured texture parameters, Pearson’s correlation statistic was calculated to estimate significant correlations between genes and physical parameters. From the co-expressing merged modules (denoted by colors), eigengene expression values were calculated and visualized to determine connections with the NR process. The correlation and significance level between texture parameters and co-expression modules were also calculated and visualized using a heatmap. The created co-expressing modules were based on eigengene expression patterns showing a resemblance to the NR process (Noble Rot Connected Modules, NRCM). Using the eigengene expression values of the NRCMs test result, an ANOVA test was performed to find significant changes between neighboring phases and dates within the NRCMs. In addition, Tukey’s HSD test was performed to test for significant increase or decrease. The significant increases and decreases of each NRCMs were selected for GO and Pathway Enrichment.

#### 2.3.3. Differential Gene Expression and Enrichment

To compare the changes in the functional gene composition among the NR phases, a serial differential expression (DE) analysis was performed between phases I and II, II and III, and III and IV respectively using the DESeq2 R package [37]. *p*-value of 0.05 and log2(fold change) of 1 were set as the threshold for significantly differentially expressed genes. Differentially expressed up- or down-regulated functional genes belonging to neighboring phases were assigned and grouped by NRCM membership. For example, in the blue module, there was a significant increase in the eigengene expression between phases I and II. Therefore, all up-regulated genes between phases I and II from the blue module were selected for GO and Pathways enrichment. This pipeline was followed for all NRCMs, using the correlation between eigengene expression and DE analyses as guide for further enrichment analyses. GO enrichment of the selected genes from the respective modules was performed using the topGO R [38] whereas pathway enrichment was performed using the KOBAS v 2.0 software tool [39]. Term and pathways with corrected *p*-values < 0.05 were considered significantly enriched.

## 3. Results

### 3.1. Statistical Analyses

**Figure 1:**
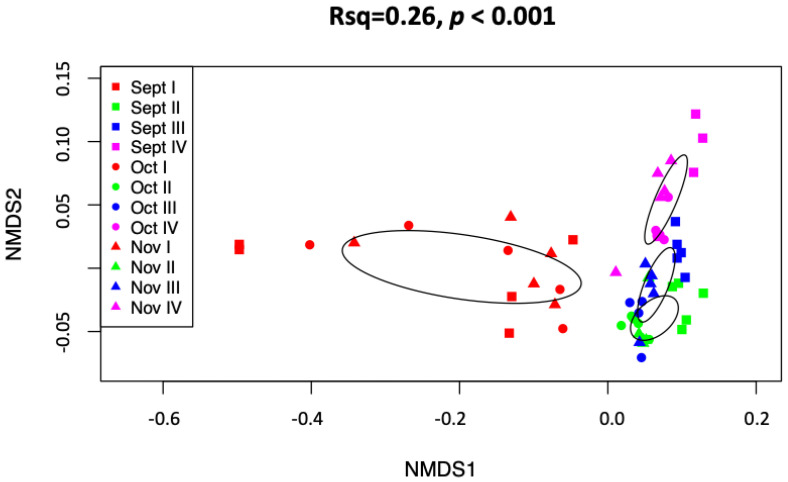
Non-metric multidimensional scaling analyses of combined September-October-November data expressed functional genes of *Botrytis cinerea* for the noble rot phases. Ellipses depicting standard deviation from the centroid of each phase show no or little overlap, indicating a strong compositional difference in expressed functional genes among botrytization process, without separation in sampling months.

The distribution of transcript abundance and richness of *B. cinerea* showed a significant increase between phase I (healthy) and all other phases (II, III, IV) of NR but there were no significant changes among the other phases. There was no significant difference in either abundance or richness between the respective sampling months (Figure 1). The ordinations of the NMDS analyses showed strong separation between the NR phases, whereas harvest time appeared to have less impact on the variation of the samples. The latter was also demonstrated by the PERMANOVA test, which indicated that 26% of variation was interpreted by phases (F = 19.712, *p* < 0.01, r^2^ = 0.26), and to months with 6.6% of variation effected by sampling time (F = 4.01, *p* < 0.01, r^2^ = 0.066).

### 3.2. Weighted Gene Co-Expression Analyses

**Figure 2:**
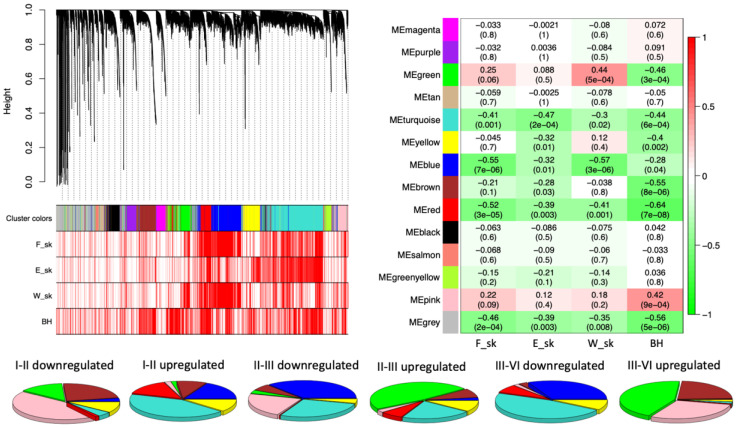
Co-expression network analysis of expressed *Botrytis cinerea* genes of four noble rot phases. A hierarchical clustering tree showing 13 modules of functional genes with 100 or higher module membership. Red bars corresponding to textural parameters indicates significant (*p* < 0.05) correlation with functional genes. On the heatmap, the correlation and the corresponding *p*-values for the 13 gene modules eigengene and textural parameters are shown. Pie-charts show the representation of the different noble rot connected modules of expressed genes corresponding to the differentially expressed genes between pairs of noble rot phases.

**Figure 3:**
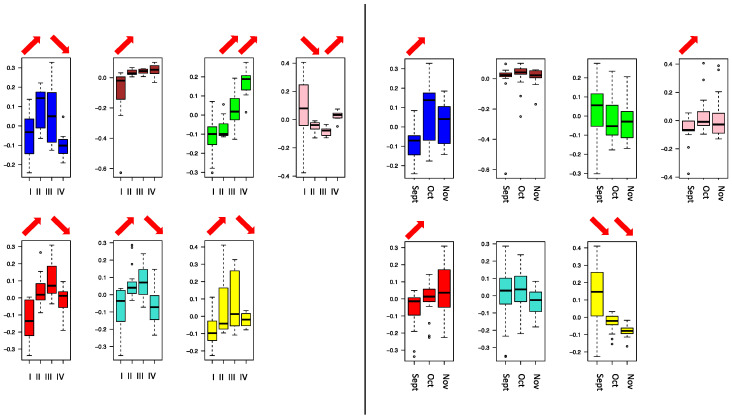
Module eigengene distributions between noble rot phases (**left**) and sampling dates (**right**) regarding to noble rot connected modules of expressed genes using one-way ANOVA models. Red arrows indicate the significant increase or decrease between neighbor phases or months.

To investigate the internal structure of the gene network, WGCNA clustering was calculated to dived co-expressing functional genes into modules denoted by colors. From the WGCNA clustering analyses, 13 different co-expression modules with more than 100 members were merged (Figure 2). It was found that berry skin break force (F_sk) has a negative correlation with the turquoise, blue, and red modules. Similarly, the berry skin elastic modules (E_sk) have significant negative correlations with the turquoise, yellow, blue, brown, and red modules. The skin break energy (W_sk) showed a significantly positive correlation with the green module but a negative correlation with the turquoise, blue, and red modules. The berry hardness showed a significantly positive correlation with the pink module but a negative correlation with the green, turquoise, yellow, blue, brown, and red modules. Among the modules, the turquoise, blue, brown, and red modules showed the most significant negative correlation with textural parameters, showed on the heatmap part of Figure 2. The blue, brown, green, pink, red, turquoise, and yellow NRCMs showed an eigengene expression profile determined by NR phases or months (Figure 3) whereas the other six modules had a distinct and unrelatable eigengene expression profile. The Tukey HSD test of *B. cinerea* gene abundance showed a significant difference between phase I and II but no significant difference between phase II, phase III, and phase IV. However, the NMDS plot on Figure 1 indicated the separation of phase samples. Within the phase I to phase II transition, 84% of the genes were clustered into modules that had a significant negative correlation with texture parameters. 

### 3.3. Differential Expression Analyses

Throughout the entire noble rot process, 8089 genes were differentially expressed among the respective phases. Between phases I and II, 1094 genes were found to be differentially expressed. These genes showed no change between phases II and III but were down-regulated from phase II to IV. A total of 440 genes, not found between any other phases, was differentially expressed between phases I and II. Between phases II and III, and III and IV, 319 and 870 unique genes were found to be up-regulated. With regards to down-regulation, the only scenario in which there was a significant difference was the 2932 down-regulated genes between phases III and IV. A total of 1126 genes were found to be down-regulated at the early stages of the noble rot process (between phases II and III). Finally, 344 genes were found to be down-regulated at the early NR stages, but then up regulated in the last stage. Table 2 shows the total and unique up- and down-regulated genes of the serial differential expression analyses.

### 3.4. Gene Ontology and Pathway Enrichment

Several enriched GO’s and pathways were identified in all significant phase transitions of all NRCMs. Among these, the most were associated with carbohydrate and protein metabolism, both of which play an important role during the NR process (Table 3). Among the 17 enriched pathways from the brown module phase I to II up-regulated genes, “carbon metabolism”, “fructose and mannose metabolism”, “glycolysis”, and “butanoate metabolism” were identified. In the red module, up-regulated genes from phase I to II were significantly enriched for “starch and sucrose metabolism”. In the turquoise module, up-regulated genes from phase I to II were enriched for “galactose metabolism”, “glycolysis”, “other glycan metabolism”, and “starch and sucrose metabolism”. Carbohydrate metabolism was also found to be enriched between phase III and IV at the end of the botrytization process, where up-regulated genes in green module were enriched for “carbon metabolism” and “glycolysis”, and for “carbon metabolism”, and “pyruvate metabolism” in the pink module.

Protein metabolism-related enriched pathways were found in the up-regulated genes from phase I to phase II of the brown module such as “alanine and aspartame metabolism”, “tryptophane metabolism”, ”valine, leucine, and isoleucine metabolism” as well as “ubiquitin mediated proteolysis”. In addition to pathways pertaining to amino acid metabolism, the “ketone bodies” pathway was also found to be enriched in the up-regulated brown module phase I to II genes. With regards to the green module, enriched pathways pertaining to protein metabolism, such as the “alanine and aspartame metabolism”, “beta-alanine”, “cysteine and methionine metabolism” and “glycine, serine, and threonine” were identified as being enriched during the later phase III and IV of the noble rot processes.

In addition to carbohydrate and protein metabolism, most of the modules showed pathways responsible for the formation of secondary metabolites. These compounds play a role in NR development such as aromatic compound formation and antibiotic activity. For example, in the pink module, both significant phase transitions (genes up regulated from phase I to II and II to III respectively) contained significantly enriched pathways associated with “sulfur metabolism”. In the brown module, the “alkaloid biosynthesis” pathway was found to be enriched among the up-regulated genes from phase I to II. The same pathway was found to be enriched in the up-regulated genes between phase III and IV in the green module. The entire list of enriched pathways can be found in Supplementary File S1.

## 4. Discussion

The differences in certain visual and measurable physico-chemical properties of NR berries that had been used define the distinct phases of the NR process [9,14] implicitly implying that these changes were caused at least partly by a multitude of expressed functional genes of *B. cinerea* that were still unknown at the time. To our knowledge, this is the first study to shed light on the qualitative and quantitative differences in the functional gene profile of *B. cinerea* throughout the development of naturally occurring NR in field conditions, and how these functional genes likely contribute to the physico-chemical changes in botrytized grape berries, with important implications for winemakers. In oenological practice, berry texture is an important factor, because it greatly influences maceration, which, in turn, determines the release of polyphenols in berries. The polyphenol content of wines has a major impact not only on their organoleptic properties but also on their shelf life. While the degradation of the berry skin by *B. cinerea* causes a decline in polyphenol content in the skin itself, the plant produces various polyphenols as a response to the infection, resulting in a specific polyphenol composition during NR [1,5,15,25].

### 4.1. Expression Dynamics of Functional Genes of Botrytis cinerea during Noble Rot

The fact that functional gene transcripts of *B. cinerea* significantly increase in abundance and richness from the first to the second phase but stays consistent thereafter indicates a major physiological shift of *B. cinerea* characterized by a surge of metabolic activity at the onset of NR, which confirms our first hypothesis (i). The significant increase in the richness and abundance functional gene transcripts of *B. cinerea* from healthy to NR berries agrees with previous results in artificially induced postharvest NR [23], whereas the current study, where all NR phases were investigated, reveals that the increase in expressed functional genes already occurs during the onset of the NR process. The major up-regulation of *B. cinerea* functional genes early in the NR process, reported here, confirms that *B. cinerea* is mostly inactive in healthy, mature berries [23]. As the NR process starts, presumably in response to environmental cues, it becomes metabolically more active, and expresses genes involved in proliferation, and genes that initiate NR-associated changes to *V. vinifera* such as breakdown of the berry skin [9], and activation of plant defense responses and ripening [40]. The overall similarity of the *B. cinerea* noble rot gene expression profile across the respective months shows that the composition of expressed genes of *B. cinerea* in NR is temporally relatively stable, which implies a certain predictability. The latter was also confirmed by the NMDS analyses which showed a clear separation of phase II, III, and IV when all months were combined, which confirms our second hypothesis (ii). The latter is of crucial importance to winemakers.

### 4.2. Botrytis cinerea Functional Genes Involved in Berry Skin Degradation during Noble Rot

*Vitis vinifera* skin consists of mainly carbohydrate-based compounds such as cellulose, arabinan, mannan, galactan, xylan, and xyloglucan, lignin structural proteins, and proanthocyanidins [41,42]. We identified numerous differentially expressed genes within NRCM modules that significantly correlated with texture parameters, which indicate their importance in the characteristic degradation of the berry skin during the botrytization process. It should be noted that the NR is a serial development consisting of four phases which lends itself to comparing neighboring phases and months in each type of analysis. According to the ANOVA and Tukey’s tests based on the eigengene expression of the WGCNA clustered modules that followed a NR based pattern, it was found that genes that were differentially expressed during the transition from healthy to initial NR berries (from phase I to phase II) were the most relevant with regards to typical *aszú* development [1,9,43]. This was due to the observation of a significant “E-drop” for the elastic modulus of the berry skin at that stage, which indicates that cell wall decomposition mainly occurs during this early stage of NR. The clustering of carbohydrate metabolizing enzymes to berry skin physical parameters in the current study confirms their active role in contributing to the textural changes that take place during NR. Carbohydrate active (CA) enzymes, that are involved in carbohydrate synthesis and/or degradation either directly or indirectly, are of particular interest with regards to berry skin degradation. We identified several differentially expressed CA enzymes between phases I and II, i.e., corresponding to the onset of NR, in the brown, red, and turquoise modules. This confirms our hypothesis (iii) that cell wall catabolic enzymes are of particular importance in the early phases of the NR process. The CA enzymes identified in the brown module could be related to preliminary softening of the berry skin, as these are mostly indirectly involved in carbohydrate degradation processes. An example of such an enzyme is glucose-6-phosphase isomerase (BCIN_15g04970), which is involved in the sub-pathway of glycolysis [44,45]. In contrast to the brown module, the red and turquoise module contained enzymes that are directly involved in carbohydrate degradation, cleaving bonds within the carbohydrate polymer structures. For example, alpha-amylase (BCIN_02g01420) cleaves polymeric substrate into shorter oligomers catalyzing the hydrolysis of alpha-1,4-glucane linkages [46,47]. Most of these enzyme types were found in the turquoise module which has the highest negative correlation with the skin elasticity, which indicates the beginning of the NR process [9].

### 4.3. Botrytis cinerea Gene Functions in Other Biochemical Processes in Noble Rot

During NR, several biochemical changes take place which have been thoroughly described in literature [15,43,48]. For example, it has been found that the sugar content increases in botrytized berries. The latter can reach levels as high as 350–700 g/L in *Tokaj aszú* berries [1,9,15]. In addition to causing structural changes to the berry skin, carbohydrate degrading enzymes as described above can also lead to an increase in sugar content. The current study highlights that carbohydrate degrading enzymes are mostly active during the early NR stages. In addition to carbohydrate metabolism, enriched pathways involving protein metabolism was also predominant in the respective modules. In addition to contributing to the structural importance of *V. vinifera* [49], nitrogen-containing compounds such as amino acids, peptides, and nucleic acid derivatives play an important role for *B. cinerea* during the NR process. For example, it uses nitrogen sources for several metabolic pathways during infection [48]. This possibly explains the identification of the nitrogen liberating enzymes such as omega-amidase (BCIN_14g01750) as part of the enriched alanine-aspartame pathway [50] up-regulated between phase I and II in the brown module and glutamate dehydrogenase (BCIN_03g07670) belonging to the enriched alanine-aspartame pathway up-regulated between phase III and IV in the green module. The identification of the enriched pathway “ketone bodies” also suggests the degradation of amino-acids containing ketone bodies. The current study shows the importance of proteins in the structural changes that take place in NR berries. In addition, it demonstrates the active role of *B. cinerea* in liberating nitrogen during NR, but also demonstrates that this phenom already occurs during the early stages of NR.

### 4.4. Functional Genes Associated with Secondary Metabolites

It has been demonstrated that *B. cinerea* is the predominant fungus during NR [51,52,53,54,55], which suggests a certain competitive advantage over other microorganisms in the microenvironment of the NR grapes characterized by increasing osmotic stress resulting from water loss and sugar content increase. In addition, in viticultural practices, sulfur metabolism plays an important role as sulfur-containing compounds are sprayed onto grapevines to lower the incidence of plant pathogens. High levels of these compounds can, however, accumulate on the berries to levels that are toxic to *B. cinerea* as well as other fungi. Sulfur metabolism and subsequent detoxification are, therefore, of importance in the plant–fungi interaction model during the NR process [56]. In the pink module, the sulfur metabolic pathway was enriched between phases I and II and III and IV respectively. In the early phases (I to II), three down-regulated enzymes belonging to the enriched sulfate pathway namely sulfate adenylyltransferase (BCIN_01g04340), phosphoadenosine phosphosulfate reductase (BCIN_01g04230), and sulfite reductase (BCIN_13g03450) were identified which catalyze the reduction from prosphoadenosin phosphosulface (PAPS), PAPS to sulfite and sulfite to hydrogen sulfide respectively [57], whereas in the later NR phases (III to IV) phosphoadenosine phosphosulfate reductase (BCIN_01g04230), sulfite reductase (NADPH) hemoprotein beta-component (BCIN_12g03740), and O-acetylhomoserine/O-acetylserine sulfhydrylase (BCIN_04g0077) belonging to the sulphate enriched pathway convert PAPS to sulfite, sulfite to sulfide, sulfide to L-cysteine respectively were up-regulated. Interestingly, in the yellow cluster, the glutathione metabolism pathway (bfu00480) of *B. cinerea* was highly significantly up-regulated during the early NR stages (I and II), but the pathway was down-regulated during the later stages (III and IV). As mentioned previously, sulfite can be decomposed to L-cysteine via the sulfur metabolic pathway. The latter compound can be converted to glutathione at a high rate via the cysteine and methionine metabolic pathway (bfu00270). The latter compound has important functions in cellular functions, mitochondrial structure, cell differentiation as well as cell development [16]. In addition, it can play an important role in biotic and abiotic oxidative stress responses such as heat shock and osmotic stress [58,59,60]. *B. cinerea* can reduce sulfur compounds, thereby lowering the quantities of these compounds to levels that are suited to its survival. Furthermore, it can transform these compounds to other secondary metabolites that are beneficial for its proliferation. In addition to *B. cinerea* producing and transforming metabolites in favor of its proliferation, compounds that have antagonistic activity to other microorganisms were identified. In the brown module, the enzyme catalase (BCIN_03g01920) belonging to the enriched “secondary metabolites” pathway up-regulated between phases I and II was identified. This enzyme forms part of several enriched pathways, such as the carbon and glyoxylate enriched pathway, but is also a key enzyme in the synthesis of alkaloids. The latter group contains compounds that have antagonistic activity such as the siderophore ferricrocin [61]. In the green module, the enzymes 3-dehydroquinase (cDHQase) (BCIN_09g00160) and 5-aminolevulinate synthase (BCIN_10g03090) as belonging to the enriched “secondary metabolites” pathway up-regulated between phases III and IV were identified. The enzyme cDHQ is responsible for the synthesis of 3,4-dihydroxybenzoate via the shikimate-pathway and is a marker of *B. cinerea* infection with known antagonistic properties toward other microorganisms [62,63,64]. The enzyme 5-aminolevulinate synthase (BCIN_10g03090) can form porphyrin-containing compounds such as protoporphyrin-IX, which is also expected to have antagonistic activity, as the use of porphyrin-containing compounds as alternatives to antibiotics has been demonstrated already [65,66,67]. These compounds are effective in vitro in the photodynamic inactivation of bacteria, viruses, fungi, and protozoa based on the formation of reactive oxygen species [68,69]. It is important to note that the porphyrin-containing compounds produced by *B. cinerea* may also damage the fungus itself. However, this enzyme may also play a role in adapting to changes in environmental factors, such as high sugar levels, through heme synthesis [70] and further studies are needed to clarify the roles of porphyrin-containing compounds in the NR process. The antagonistic activity of *B. cinerea* throughout the early and late NR phases contributes to its ability to dominate during NR. The ability of *B. cinerea* to produce antagonistic elements during the later NR was recently poorly studied. The production of antimicrobials by *B. cinerea* confirms the final hypothesis (iv), confirming that this species has adaptive properties that allow it to dominate during NR.

## 5. Conclusions

The NR process is based on a complex interaction between *V. vinifera* and the most predominant fungus in NR grape berries, *B. cinerea*. This study provides detailed insights into the functional roles of *B. cinerea* in the development of the four respective NR phases, particularly pertaining to the textural changes of the grape berry. Our data suggest that *B. cinerea* has a relatively predictable succession of changes in expression profile during the development of NR that is not substantially affected by the month. WGCNA clustered modules in the current study were mainly enriched for pathways pertaining to protein and carbohydrate metabolism, highlighting their importance in contributing to the grape berry structure. The WGCNA clustering and analyses of modules that had an eigengene expression profile relatable to the NR process indicated that *B. cinerea* mainly contributes to NR textural changes during the initial NR phases (I and II) as most enriched pathways pertaining to carbohydrate degradation were expressed during these two phases. The degradation of the berry skin and hardness of the whole berry are indicators of the quality of NR, which can be used by viticulturists to identify the phase of botrytization and *aszú* development. In addition to the identification of *B. cinerea* genes that contribute to the structural changes that take place during NR, the study also highlighted the presence of genes that allow it to proliferate and dominate in its environment such as the ability to metabolize sulphate in its environment, and to produce antimicrobials, which allows it to dominate.

## Figures and Tables

**Table 1:** Average and standard deviation values of textural parameters corresponding to each noble rot phase. Letters indicate the significant differences in post-hoc Tukey’s test (*p* < 0.05).

Phase	F_sk_ [N]	E_sk_ [N/mm]	W_sk_ [mJ]	BH [N]
I	0.321 ± 0.043 a	0.345 ± 0.071 a	0.208 ± 0.011 a	3.979 ± 0.149 a
II	0.156 ± 0.057 b	0.249 ± 0.054 b	0.129 ± 0.082 a	1.005 ± 0.128 b
III	0.134 ± 0.033 b	0.204 ± 0.010 b	0.124 ± 0.053 a	0.681 ± 0.127 c
IV	0.364 ± 0.256 a	0.357 ± 0.167 a	0.352 ± 0.248 b	1.190 ± 0.675 b

**Table 2:** Distribution of differentially expressed functional genes between noble rot phases. Upward arrows indicate up-regulation, downwards indicates down-regulation, zero indicates no difference in expression level regarding to phase transitions.

Phase I–II	Phase II–III	Phase III–IV	Number of DE-ed Genes
↑	↑	↑	3
		⌀	25
		↓	19
	⌀	↑	41
		⌀	440
		↓	1094
	↓	↑	3
		⌀	26
		↓	50
⌀	↑	↑	167
		⌀	319
		↓	94
	⌀	↑	870
		⌀	n.r.
		↓	2932
	↓	↑	20
		⌀	110
		↓	137
↓	↑	↑	33
		⌀	35
		↓	5
	⌀	↑	344
		⌀	1126
		↓	161
	↓	↑	8
		⌀	13
		↓	23

**Table 3:** List of enriched Gene Ontologies corresponding to noble rot connected modules in statistically significant phase transitions.

Blue I–II Up-Regulated	*p*-Value	Pink I–II Down-Regulated	*p*-Value
peptidase activity	<0.001	organic cyclic compound binding	<0.001
serine-type peptidase activity	<0.001	heterocyclic compound binding	<0.001
serine hydrolase activity	<0.001	RNA binding	<0.001
catalytic activity, acting on a protein	<0.001	nucleic acid binding	<0.001
hydrolase activity	<0.001	binding	0.007
carboxypeptidase activity	<0.001	DNA-directed 5′-3′ RNA polymerase activity	0.014
serine-type carboxypeptidase activity	<0.001	5′-3′ RNA polymerase activity	0.014
serine-type exopeptidase activity	<0.001	RNA polymerase activity	0.014
exopeptidase activity	0.002	aspartic-type endopeptidase activity	0.035
catalytic activity	0.004	aspartic-type peptidase activity	0.035
endopeptidase activity	0.007	purine ribonucleoside triphosphate binding	0.041
aspartic-type endopeptidase activity	0.015	N-methyltransferase activity	0.043
aspartic-type peptidase activity	0.015	arginine N-methyltransferase activity	0.043
ubiquitin-like protein-specific protease activity	0.026	protein-arginine N-methyltransferase activity	0.043
NEDD8-specific protease activity	0.026		
		**Red I–II Up-Regulated**	***p*-Value**
**Blue III–IV Down Egulated**	***p*-Value**	transferase a., transferring glycosyl group	0.018
serine-type peptidase activity	<0.001	NAD + ADP-ribosyltransferase activity	0.035
serine hydrolase activity	<0.001	transferase a., transferring pentosyl groups	0.035
peptidase activity	<0.001		
hydrolase activity	<0.001	**Red III–IV Down-Regulated**	***p*-Value**
catalytic activity	<0.001	phasphatase activity	0.001
carboxypeptidase activity	0.003	phophoric ester hydrolase activity	0.001
serine-type carboxypeptidase activity	0.003	protein tyrosine phophatase activity	0.007
serine-type exopeptidase activity	0.003	hydrolase activity, acting on ester bonds	0.013
hydrolase a., acting on carbon-nitrogen bond	0.006	phosphoprotein phosphatase activity	0.019
exopeptidase activity	0.009		
serine-type endopeptidase activity	0.012	**Turquoise I–II Up-Regulated**	***p*-Value**
catalytic activity, acting on a protein	0.013	cation binding	<0.001
DNA-binding transcription factor activity	0.020	metal ion binding	<0.001
transcription regulator activity	0.041	phosphorelay sensor kinase activity	<0.001
		protein kinase activity	<0.001
**Brown I–II Up-Regulated**	***p*-Value**	protein histidine kinase activity	<0.001
antioxidant activity	<0.001	phosphotransferase activity, nitrogenous groups	<0.001
oxidoreductase activity, acting on peroxidase	<0.001	small molecule sensor activity	<0.001
peroxidase activity	<0.001	kinase activity	0.001
fatty-acyl-CoA binding	0.013	phosphotransferase activity, alcohol groups	0.003
hydroxymethylglutaryl-CoA lyase activity	0.013	ion binding	0.007
alanine-glyoxylate transaminase activity	0.013	binding	0.010
transaminase activity	0.013	transferase activity, transferring phosphorous groups	0.011
transferase activity, transferring nitrogen	0.013	catalytic activity, acting on a protein	0.017
oxo-acid-lyase activity	0.013	cytoskeletal protein binding	0.029
peroxiredoxin activity	0.013	ADP binding	0.041
acyl-CoA binding	0.013	double-stranded DNA binding	0.047
fatty acid derivative binding	0.013	double-stranded telomeric DNA binding	0.047
sulfur compound binding	0.013	thiol-dependent ubiquitin-specific protease	0.047
carbon-carbon lyase activity	0.026	microtubule binding	0.047
acetyl-CoA C-acyltransferase activity	0.038	omega peptidase activity	0.047
C-acyltransferase activity	0.038	tubulin binding	0.047
catalytic activity	0.047	telomeric DNA binding	0.047
		ubiquitinyl hydrolase activity	0.047
**Green II–III Up-Regulated**	***p*-Value**		
4 iron, 4 sulfur cluster binding	0.026	**Turquoise III–IV Down-Regulated**	***p*-Value**
proteasome binding	0.026	cation binding	<0.001
		metal ion binding	<0.001
**Green III–IV Up-Regulated**	***p*-Value**	binding	<0.001
double-stranded RNA binding	0.003	ion binding	<0.001
oxidoreductase activity, acting on a sulfate	0.003	phosphotransferase activity, alcohol groups	<0.001
oxidoreductase activity	0.013	kinase activity	<0.001
peptidyl-prolyl cis-trans isomerase a.	0.046	phosphorelay sensor kinase activity	<0.001
cis-trans isomerase activity	0.046	protein kinase activity	<0.001
		protein histidine kinase activity	<0.001
**Pink III–IV Up-Regulated**	***p*-Value**	phosphotransferase activity, nitrogenous groups	<0.001
RNA binding	<0.001	small molecule sensor activity	<0.001
nucleic acid binding	<0.001	transferase activity, transferring phosphorous groups	<0.001
organic cyclic compound binding	<0.001	ADP binding	0.003
heterocyclic compound binding	<0.001	GTPase activator activity	0.004
binding	0.003	enzyme activator activity	0.004
catalytic activity, acting on RNA	0.004	GTPase regulator activity	0.006
DNA-directed 5′-3′ RNA polymerase activity	0.017	nucleoside-triphosphatase regulator activity	0.006
endoribonuclease activity	0.017	molecular function regulator	0.006
ribonuclease activity	0.017	enzyme regulator activity	0.008
endoribonuclease activity, producing 5′-phosphomonoesthers	0.017	phospholipid binding	0.010
5′-3′ RNA polymerase activity	0.017	phosphatidylinositol binding	0.010
3′-tRNA processing endoribonuclease activity	0.017	protein dimerization activity	0.012
RNA polymerase activity	0.017	protein binding	0.013
endonuclease a., active with ribo- and dezoxyribo-nucleic acid	0.033	cytoskeletal protein binding	0.019
endonuclease activity	0.050	adenyl nucleotide binding	0.039
N-methyltransferase activity	0.050	adenyl ribonucleotide binding	0.039
arginine N-methyltransferase activity	0.050	zinc ion binding	0.044
protein-arginine N-methyltransferase activity	0.050	transition metal ion binding	0.044
**Yellow III–IV Down-Regulated**	***p*-Value**	**Yellow I–II Up-Regulated**	***p*-Value**
FMN binding	0.006	aspartic-type endopeptidase activity	0.009
hydrolase activity, acting on ether bonds	0.064	aspartic-type peptidase activity	0.009
		transferase activity, transferring acyl groups	0.022
		endopeptidase activity	0.027
		peptidase activity	0.048

## Data Availability

DNA sequences have been deposited in NCBI under BioProject PRJNA736205 and BioSAMPLE SAMN19612984. https://www.ncbi.nlm.nih.gov/bioproject/.

## Supplementary Materials

The following supporting information can be downloaded at: https://www.mdpi.com/article/10.3390/jof8040378/s1, Supplementary File S1. The entire list of enriched *B. cinerea* metabolic pathways.
